# Combination of electromagnetic navigation bronchoscopy-guided biopsy with a novel staining for peripheral pulmonary lesions

**DOI:** 10.1186/s12957-019-1704-7

**Published:** 2019-09-10

**Authors:** Kai Qian, Yi Deng, Cheng Shen, Yong-Geng Feng, Bo Deng, Qun-You Tan

**Affiliations:** 1 Department of Thoracic Surgery, Institute of Surgery Research, Daping Hospital, Army Medical University, Chongqing, China; 2 Department of Oncology, Institute of Surgery Research, Daping Hospital, Army Medical University, Chongqing, China

**Keywords:** Electromagnetic navigation bronchoscopy, Biopsy, Peripheral pulmonary lesions, Staining

## Abstract

**Background:**

The diagnosis of peripheral pulmonary lesions (PPLs) is a challenging task for pulmonologists, especially for small PPLs. Conventional localization of these small PPLs, which are > 1 cm away from the visceral pleura in operation, is quite difficult. Currently used methods inevitably damage the visceral pleura and may cause a series of complications, such as pneumothorax and hemothorax. Hence, the present study aimed to find out an intraoperative localization method with no damage to the visceral pleura.

**Methods:**

We retrospectively reviewed 21 patients with PLLs who underwent electromagnetic navigation bronchoscopy (ENB)-guided biopsy plus a new methylene blue staining with the help of massage (Massage Staining) in our department between August 2017 and December 2018.

**Results:**

The median age of these 21 patients was 51.3 ± 2.1 years. The diameter of the PPLs was 8.2 ± 2.3 mm. The rate of successful biopsy was 76.2%, and the rate of excellent or satisfactory of Massage Staining was 81.0%, while all lesions of these 21 cases were included in the range of staining, and the median distance from the edge of the stained site to the edge of the lesion was 29 ± 18 mm. The duration of ENB-guided biopsy plus Massage Staining was 26.7 ± 5.3 min, and the intraoperative blood loss was 3.3 ± 1.5 ml. No pneumothorax, hemorrhage, and tracheal injury occurred intraoperatively.

**Conclusions:**

The ENB-guided biopsy combined with Massage Staining is an innovative one-stop strategy designed to enhance the precision of thoracic surgery. The Massage Staining avoids damage to the visceral pleura, causes the low incidence of complications, but yields precise localization of PPLs.

## Introduction

With the widespread application of computed tomography (CT), more cases with peripheral pulmonary lesions (PPLs), e.g., ground-glass nodules (GGNs), have been detected. Superficially localized solid nodules with pleural indentation can be visualized or palpable during surgery. However, the detection of pure GGNs and sub-solid nodules (SSNs) accompany with challenges. During operation, the approximate location of the pulmonary nodules can be determined by preoperative CT scan images. However, there is often a deviation in terms of location of lesions before and after the collapse of the lungs, causing that lesions cannot be accurately located, leading to tremendous medical risk. Other conventional positioning methods include CT-guided implantation of hookwire or coils and CT-guided methylene blue staining. Nevertheless, those methods damage the visceral pleura and induce pneumothorax, hemothorax, detachment, or movement from the localized object s[1].

Electromagnetic navigation bronchoscopy (ENB) is a promising technology that increases the diagnostic accuracy of peripheral lung and mediastinal lesions and is taken as a supplement to traditional bronchoscopy, endotracheal ultrasound, and endotracheal biopsy techniques into consideration [2]. Preoperative pathological biopsy by using synchronized ENB contains significant clinical values for surgical treatment of pulmonary nodules.

In this study, we initially and innovatively conducted a novel Massage Staining without damage to the visceral pleura, combining with ENB-guided biopsy for diagnosis of PPLs.

## Methods

### Patients’ selection

Patients were screened between August 2017 and October 2018 to undergo ENB-guided biopsy and Massage Staining. The inclusion criteria were as follows: (1) patients who did not undergo pathological diagnosis and anti-tumor treatments before operation; (2) being resistant to ENB-guided biopsy; (3) the distance from the edge of the lesion to the visceral pleura ≤ 15 mm; (4) patients who did not have any contraindications for pulmonary surgery, i.e., distant metastasis, bleeding tendency, blood clotting disorders, cardiopulmonary insufficiency, severe arrhythmia or hypertension, pulmonary hypertension, and acute respiratory infection. This study was approved by the Ethics Committee of the Daping Hospital, Army Medical University (Chongqing, China). Besides, all the patients signed a written informed consent form.

### Instruments

The Super-D electromagnetic navigation system was purchased from Covidien (AAS000161-02; USA), including a 1.9 × 1070-mm edge positioning guide wire, a 2.8 × 1050-mm extended working channel for the locatable guide, 1.8 × 1050-mm biopsy forceps, and a 1.8 × 1000-mm hollow cannula. Video-assisted thoracoscopic surgery (VATS) was performed using the STORZ thoracoscopic system (KARL STORZ, Culver, CA, USA), and the robotic surgery was conducted by using the da Vinci Surgical System (Intuitive Surgical Inc., Sunnyvale, CA, USA).

### Surgical procedure

**Fig. 1:**
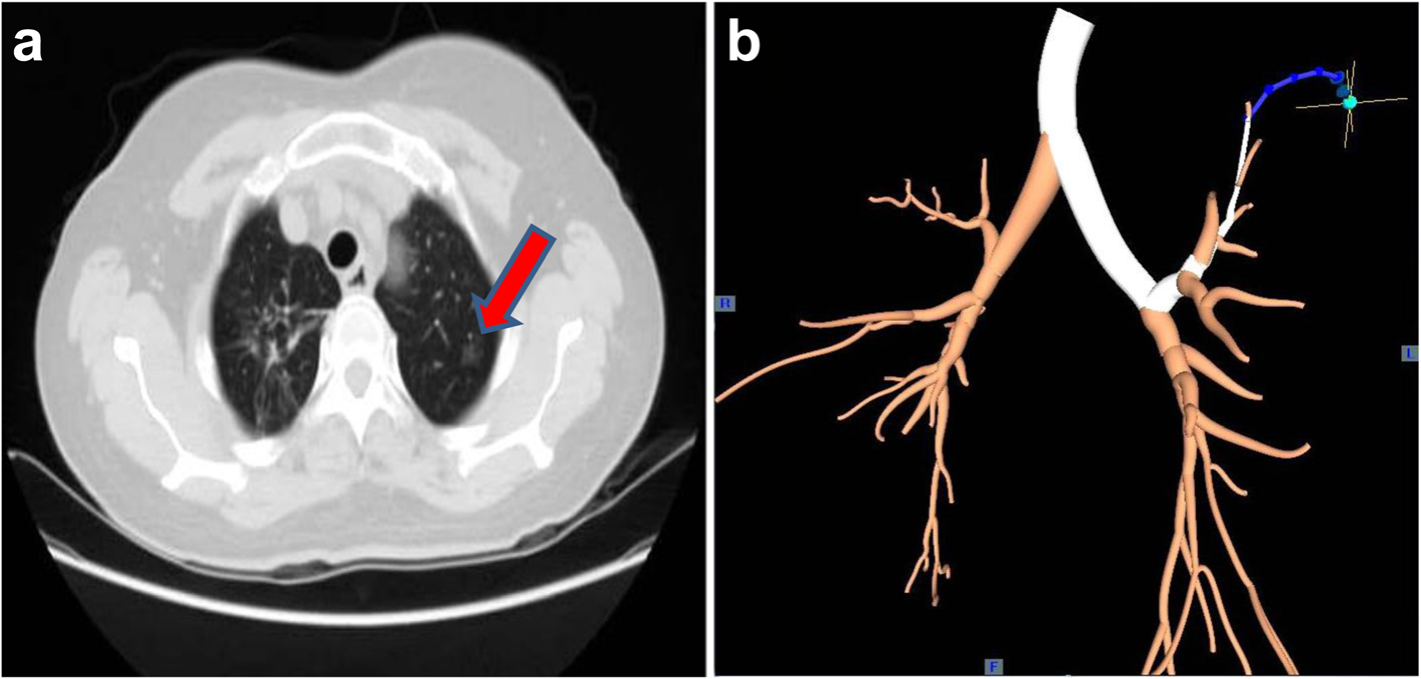
Preoperative preparation. **a** Preoperative CT scan was carried out to determine the location of the lesion; the red arrow shows the lesion. **b** Preoperatively establish the navigation path by ENB, in which the green point is a lesion

**Fig. 2:**
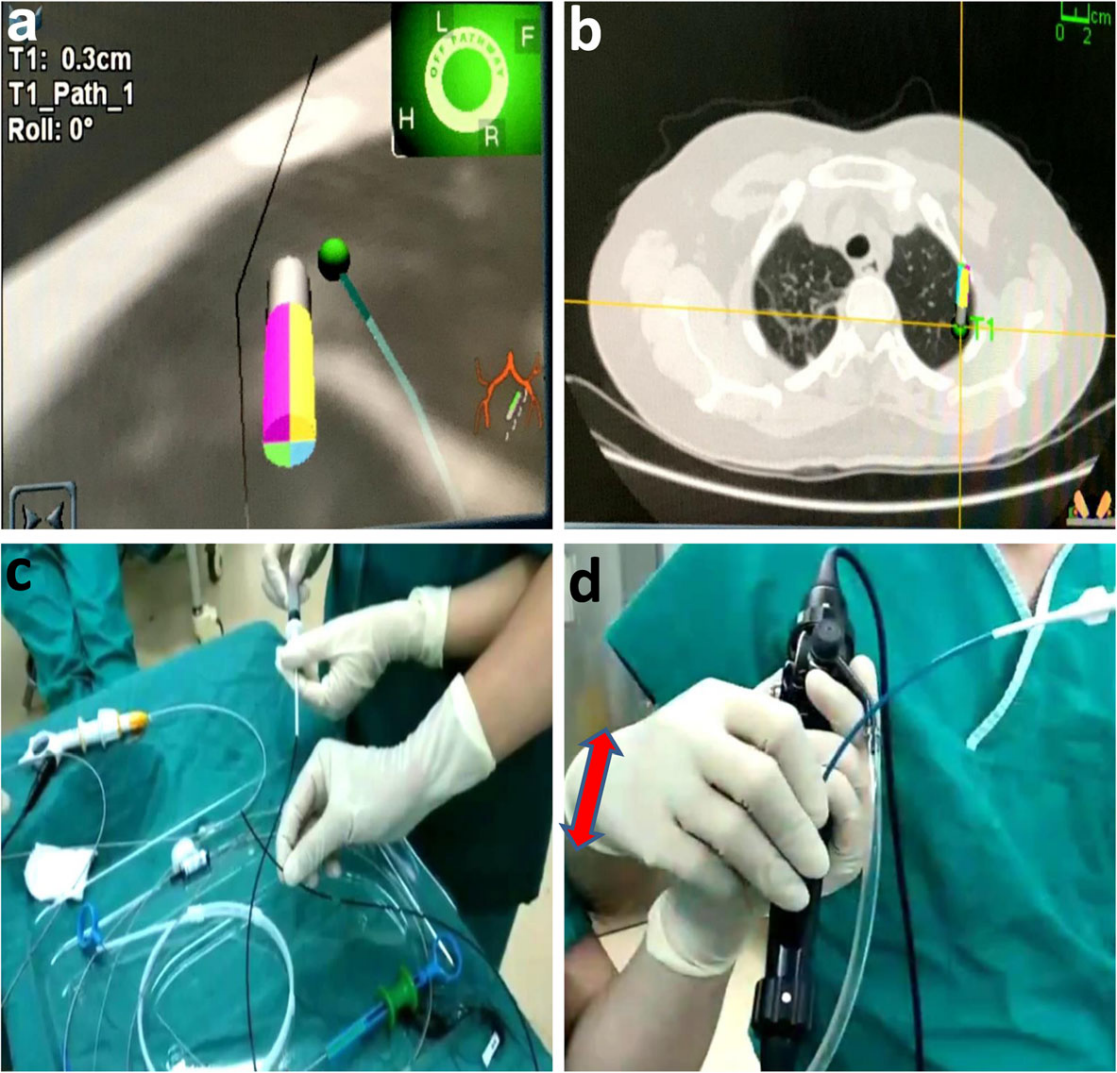
Surgical procedure for ENB-guided biopsy combined with massage staining. **a** The locatable guide wire and the extended working channel reached the lesion. **b** The locatable guide wire and the extended working channel reached the visceral pleura. **c** The methylene blue was injected through cannula with the dose of 0.8 ml/cm of the lesion diameter. **d** The catheter with methylene blue was placed into the extended working channel, and the direction of catheter rubbing during the staining was shown by the red arrow

**Fig. 3:**
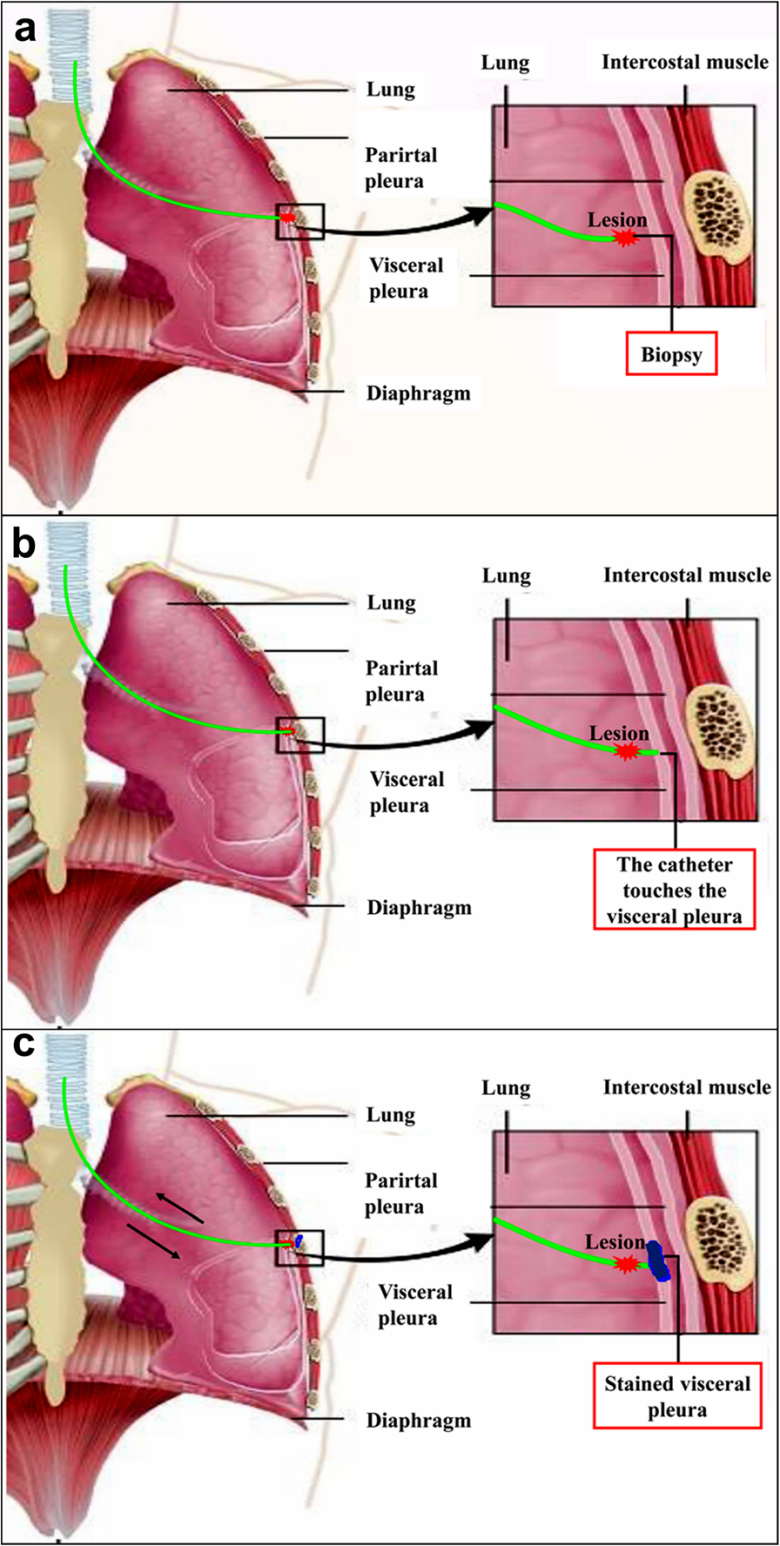
**a**–**c** Schematic diagram of a novel Massage Staining

**Fig. 4:**
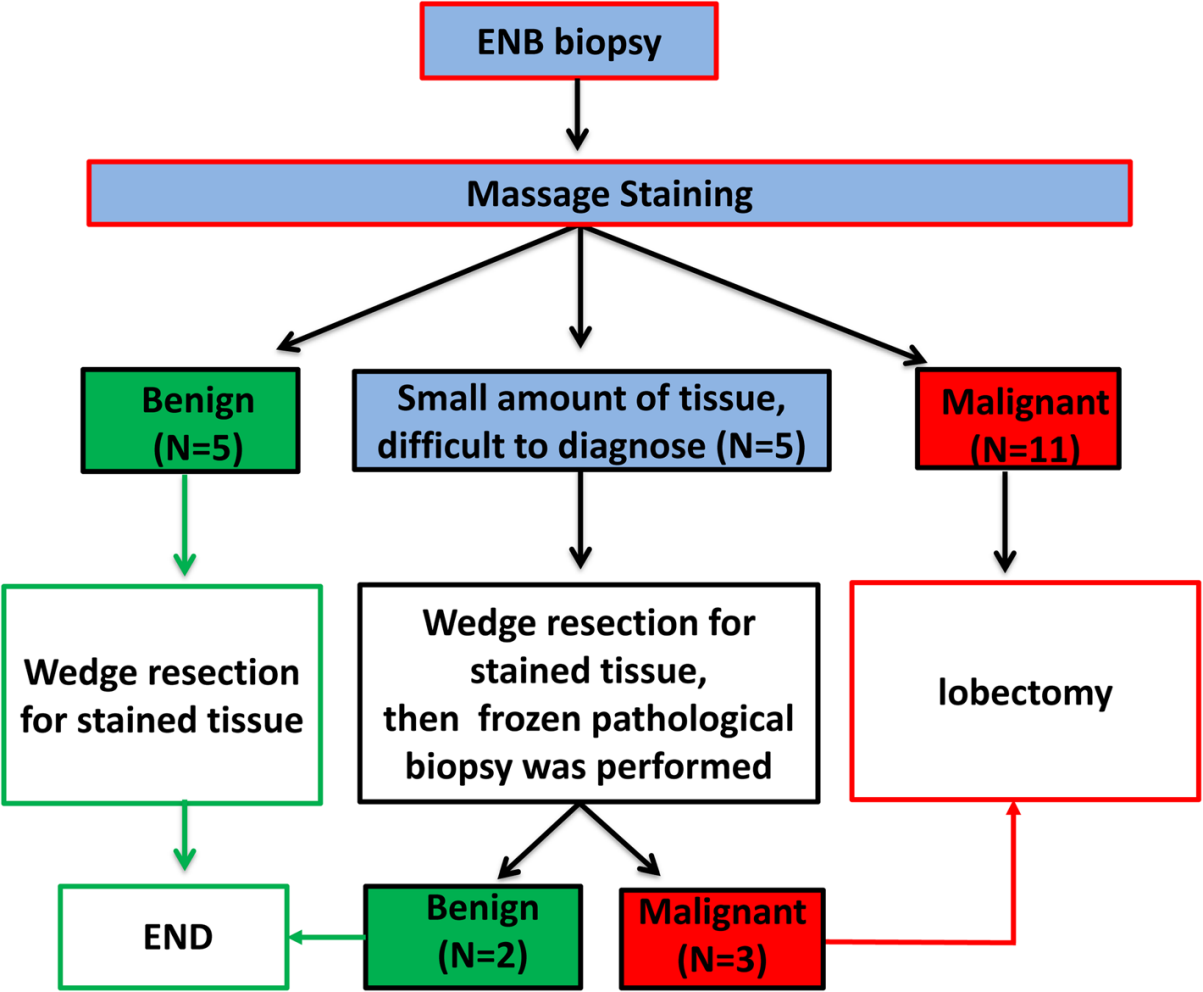
The proposed strategy for surgical treatment by a combination of ENB-guided biopsy with Massage Staining. The red arrow represents the lesion is malignant according to the results of frozen section diagnosis

The patients received general anesthesia and single-lumen intubation. After exploration with a bronchoscope, the locatable guide wire was inserted via an extended working channel to dilate the working channel, where the virtual and actual bronchoscopy images were matched by Super-D electromagnetic navigation system. A navigation map to the target area was then automatically generated (Fig. 1a, b). Using the navigation system, the position of the locatable guide wire was corrected, and the locatable guide wire was advanced to the lesion site (Fig. 2a). Subsequently, the locatable guide wire was retracted, the biopsy tool was inserted through the guide wire’s expansion channel, and the target tissue was clamped out for frozen section diagnosis. Then, the locatable guide wire was again placed to the pleural adjacent to the lesion with the guidance of the navigation system (Fig. 2b), a cannula (with diameter of 1.8 mm) equal to the length of the locatable guide wire was inserted through the extended working channel, and methylene blue was injected through the cannula (Fig. 2c) with a dose of 0.8 ml/cm per diameter of lesion. After that, the locatable guide wire was re-inserted and was confirmed to reach the presupposed location of the visceral pleura, and then, the locatable guide wire was repeatedly used to massage the visceral pleura to complete the staining process (Fig. 2d). Schematic diagram of a novel staining method using Massage Staining is shown in Fig. 3. The next surgical procedure was performed as follows (Fig. 4): (1) if the benign lesions were diagnosed by biopsy, wedge resection was undertaken; (2) lobectomy was carried out for malignant lesions; and (3) if the lesions were extremely small to underwent biopsy, performing lobectomy depended on results of frozen section diagnosis.

### Statistical analysis

Continuous variables were presented as median values. The chi-square test was applied to compare categorical variables among the groups, and *P* value < 0.05 was considered statistically significant. Statistical analyses were carried out by using SPSS 23.0 software (IBM, Armonk, NY, USA).

## Results

A total of 21 patients with PPLs [16 males (76.2%) and 5 females (23.8%); the patients’ mean age, 51.3 ± 2.1 years] underwent ENB-guided biopsy combined with the Massage Staining. There were 8 (38.1%) cases with solid nodules, 8 (38.1%) cases with mixed ground-glass nodule (mGGN), 3 (14.3%) cases with pure ground-glass nodule (pGGN), and 2 (9.5%) cases with cavitary lesions. The diameter of the PPLs was 8.2 ± 2.3 mm. There were 6 (28.6%) cases in the right upper lobe, 1 (4.8%) case in the right middle lobe, 4 (19.0%) cases in the right lower lobe, 5 (23.8%) cases in the left upper lobe, and 5 (23.8%) cases in the left lower lobe (Table 1).

**Table 1:** Patients’ clinical characteristics

Variables	Number of cases
Gender (male to female)	16:5
Age (years)	51.3 ± 2.1
The distance from the edge of the lesion to the visceral pleura (mm)	21 ± 8
The diameter of lesions (mm)	8.2 ± 2.3
ENB-guided biopsy	21
Mode of operation	
Thoracoscopic wedge resection	7
VAST lobectomy	12
Robotic lobectomy	2

The intraoperative biopsy results of 16 patients (11 malignant tumors and 5 benign lesions) were consistent with postoperative pathology, which accompanied the successful biopsy rate of 76.2%.

**Fig. 5:**
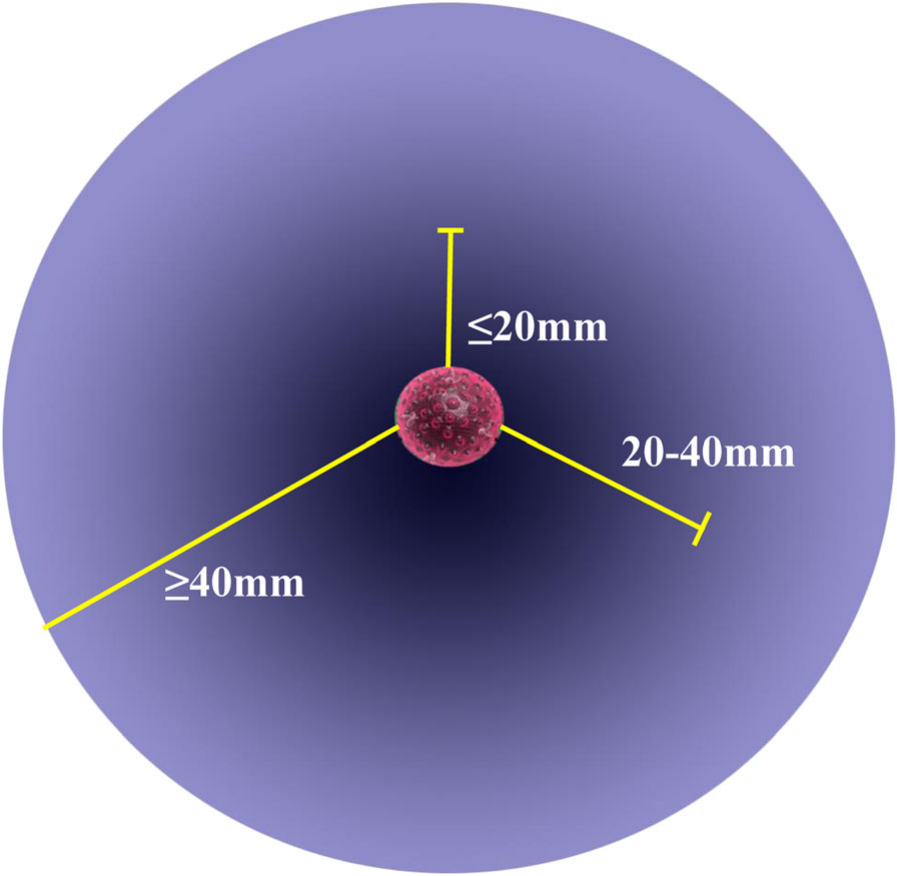
Criteria for evaluation of the dyeing effect. Excellent: the distance from the edge of the lesion to the edge of staining was less than 20 mm. Satisfactory: the distance from the edge of the lesion to the edge of staining was 20–40 mm. Unsatisfactory: the distance from the edge of the lesion to the edge of staining was more than 40 mm

**Fig. 6:**
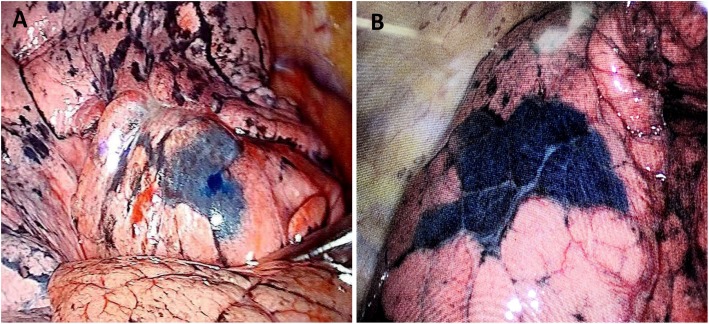
Intraoperative observation of the proposed Massage Staining. **a** The effects of staining where the catheter reached the visceral pleura. **b** The effects of staining where the distance from the catheter to the visceral pleura was about 0.8 cm

In order to assess the effects of the Massage Staining, we classified outcomes into excellent, satisfactory, and unsatisfactory, respectively (Fig. 5). The results of Massage Staining were excellent or satisfactory in 17 cases (Fig. 6a), although in the other 4 cases the result was unsatisfactory, the manipulating still guided the approximate extent of resection which is conducive to the detection of small lesions for frozen section diagnosis (Fig. 6b). The distance from the edge of the stained site to the edge of the lesions was 29 ± 18 mm. Additionally, no significant differences were noted in the results of the proposed Massage Staining method in terms of different sizes and imaging features of PPLs (*P* > 0.05) (Table 2). According to the results of frozen section diagnosis, 5 cases were diagnosed with the granulomatous or inflammatory lesions and underwent thoracoscopic wedge resection (TWR). One patient diagnosed with tuberculosis was converted to thoracotomy, and wedge resection was performed. Besides, one of them was not precisely diagnosed by ENB-guided biopsy, and according to the history of medical imaging, VATS wedge resection was carried out. Moreover, 12 patients were diagnosed with non-small cell lung cancer (NSCLC). Therefore, VATS lobectomy and lymphadenectomy were undertaken, and 2 patients received lobectomy by the da Vinci Surgical System. No pneumothorax, hemorrhage, and tracheal injury occurred during ENB-guided biopsy combined with Massage Staining. The average duration of surgery for the ENB-guided biopsy plus Massage Staining was 26.7 ± 5.3 min, and the average blood loss during the surgery was 3.3 ± 1.5 ml.

**Table 2:** Characteristics and effects of ENB-guided biopsy combined with Massage Staining for peripheral pulmonary lesions

Characteristics	Total	Dye (+)	Dye (−)	*P* value
Size of PPLs(mm)				*0.092*
≤ 9	8	6	2	
9–12	7	7	0	
12–15	5	4	1	
≥ 15	1	0	1	
Imaging features of PPLs				*0.658*
Solid nodule	8	7	1	
mGGN	8	6	2	
pGGN	3	2	1	
Cavitary nodule	2	2	0	

*Dye (+)* Excellent or satisfactory staining effects and wedge resection could be performed for the lesion; *Dye (-)* Not satisfactory staining effect with diffused dye and wedge resection was not appropriate

## Discussion

It is noteworthy that the correct diagnosis of pulmonary lesions is vital for the selection of an appropriate surgical approach. In the preoperatively diagnostic process, sensitivity and specificity of positron emission tomography-computed tomography (PET/CT) were 88% and 77%, respectively [3]. Meanwhile, the accuracy of percutaneous CT-guided pulmonary biopsy is not satisfactory, and the puncture may lead to pneumothorax and metastasis [4]. The detection rate of bronchoscopy with endobronchial ultrasound-guided transbronchial needle aspiration (EBUS-TBNA) is closely associated with the size and location of the tumor, in which the detection rate is about 63% for lesion > 2 cm, and that is reduced to 34% for lesion <  2 cm [5].

Different from the above methods, ENB employs a three-dimensional reconstruction of CT scan data and sensor location technology to guide a steerable endoscopic probe to PPLs [6]. Multicenter prospective studies have shown that the rate of successful ENB-guided biopsy can reach to 91.8% [2]. ENB-combined transbronchial lung biopsy is feasible and safe, provides larger samples, and has a higher diagnostic yield than transbronchial lung biopsy only [7].

Small peripheral pulmonary lesions (PPLs), which are 1 cm away from the visceral pleura, are usually invisible and untouchable during surgery. Conventional positioning and staining methods, e.g., CT-guided implantation of hookwire or coils, inevitably damage the pleura, potentially causing a series of complications such as pneumothorax and hemothorax. Kleedehn et al. compared methylene blue staining and hookwire localization and found that the incidence rates of complications reached 54% and 46%, respectively [8]. Our intraoperative staining localization method is very important without damaging the pleura preoperatively and associating complications. Although in 19% of cases (4/21), the locatable guide wire cannot reach the visceral pleura due to inflammation or tumor [9], resulting in dissatisfaction staining; the manipulating still guided the approximate extent of resection which is conducive to detection of small lesions for rapid frozen biopsy. The average duration of manipulation time for ENB-guided biopsy plus Massage Staining was 26.7 ± 5.3 min. However, we believe that it is necessary to take the time to obtain the frozen biopsy results and staining for the precise resection range. Furthermore, Massage Staining can save the time of detection of small lesions for rapid frozen biopsy.

In order to find out an appropriate dye marking, methylene blue, indigo carmine, and fibrin glue might be helpful. Indigo carmine dye has an advantage in that it can be visible at least 3 days after marking, while methylene blue is dissipated after several hours of staining. Therefore, indigo carmine should be the first choice for patients who cannot promptly undergo surgery after staining [10]. The mixture of methylene blue and fibrin glue has been previously reported [11]. The fibrin glue can decrease the diffusion rate of methylene blue in the airway and form a palpable blue-stained area as well [11]. However, that is relatively cumbersome and limited by materials. When fibrin glue is condensed, it is difficult to find small lesions after the operation. With regard to Massage Staining with the small dose of methylene blue, it did not produce a large diffusion area after rubbing without condensation, which enabled us to distinguish the lesions during and after surgery.

Furthermore, the details are supposed to be focused on the following: (1) The results of Massage Staining were based on the distance from staining spot to the visceral pleura, rather than size and imaging features of PPLs. (2) The dose of the dye should be as small as possible. The point-like rubbing between the cannula and the pleura can effectively mark the position of the lesion and reduce the spread of the dye.

## Conclusion

The ENB-guided biopsy plus Massage Staining is characterized by no damage to the visceral pleura, small damage to lung tissue, low incidence of complications, and high precision, as well as “one-stop” diagnosis and localization. Besides, this proposed strategy improves the rate of successful resection of PPLs in minimally invasive surgery. However, due to the small size of samples, it is essential to perform further multiple-center study to improve and optimize the proposed surgical technique.

## Data Availability

The data used and/or analyzed during the current study are available from the corresponding author on reasonable request.

## Abbreviations

CBCT: Cone-beam CT
EBUS-TBNA: Endobronchial ultrasound-guided transbronchial needle aspiration
ENB: Electromagnetic navigation bronchoscopy
GGNs: Ground-glass nodules
LND: Lymph node dissection
NSCLC: Non-small cell lung cancer
PPLs: Peripheral pulmonary lesions
TWR: Thoracoscopic wedge resection
VATS: Video-assisted thoracoscopic surgery
